# Perioperative Evaluation and Management of Patients on Glucocorticoids

**DOI:** 10.1210/jendso/bvac185

**Published:** 2022-12-02

**Authors:** Stanley M Chen Cardenas, Prasanna Santhanam, Lilah Morris-Wiseman, Roberto Salvatori, Amir H Hamrahian

**Affiliations:** Division of Endocrinology, Diabetes, and Metabolism, Department of Medicine, Johns Hopkins University School of Medicine, Baltimore, MD 21287, USA; Division of Endocrinology, Diabetes, and Metabolism, Department of Medicine, Johns Hopkins University School of Medicine, Baltimore, MD 21287, USA; Division of Endocrine Surgery, Department of Surgery, Johns Hopkins University School of Medicine, Baltimore, MD 21287, USA; Division of Endocrinology, Diabetes, and Metabolism, Department of Medicine, Johns Hopkins University School of Medicine, Baltimore, MD 21287, USA; Division of Endocrinology, Diabetes, and Metabolism, Department of Medicine, Johns Hopkins University School of Medicine, Baltimore, MD 21287, USA

**Keywords:** perioperative evaluation, perioperative management, glucocorticoids, hypothalamic-pituitary-adrenal axis suppression, glucocorticoid-induced adrenal insufficiency, adrenal crisis

## Abstract

Myriad questions regarding perioperative management of patients on glucocorticoids (GCs) continue to be debated including which patients are at risk for adrenal insufficiency (AI), what is the correct dose and duration of supplemental GCs, or are they necessary for everyone? These questions remain partly unanswered due to the heterogeneity and low quality of data, studies with small sample sizes, and the limited number of randomized trials. To date, we know that although all routes of GC administration can result in hypothalamic-pituitary-adrenal (HPA) axis suppression, perioperative adrenal crisis is rare. Correlation between biochemical testing for AI and clinical events is lacking. Some of the current perioperative management recommendations based on daily GC dose and duration of therapy may be difficult to follow in clinical practice. The prospective and retrospective studies consistently report that continuing the daily dose of GCs perioperatively is not associated with a higher risk for adrenal crises in patients with GC-induced AI. Considering that oral GC intake may be unreliable in the early postoperative period, providing the daily GC plus a short course of IV hydrocortisone 25 to 100 mg per day based on the degree of surgical stress seems reasonable. In patients who have stopped GC therapy before surgery, careful assessment of the HPA axis is necessary to avoid an adrenal crisis. In conclusion, our literature review indicates that lower doses and shorter duration of supplemental GCs perioperatively are sufficient to maintain homeostasis. We emphasize the need for well-designed randomized studies on this frequently encountered clinical scenario.

Glucocorticoids (GCs) are one of the most commonly prescribed drugs with an estimated use prevalence of approximately 1% of the US population [1]. They are very effective anti-inflammatory medications and considered first-line treatment for many autoimmune conditions. One important consequence of supraphysiological and/or long-term GC treatment is the potential for hypothalamic-pituitary-adrenal (HPA) axis suppression leading to GC-induced adrenal insufficiency (AI), which is associated with increased morbidity and mortality [2]. When associated with a stressor such as a surgical procedure, HPA axis suppression can result in adrenal crisis. This outcome was recognized in early-20th-century studies when adrenalectomized dogs experienced circulatory shock after laparotomy that could be prevented by administering GCs [3, 4]. In the 1950s, multiple reports described patients on chronic GC therapy for rheumatoid arthritis who died shortly after orthopedic surgery. Postmortem examinations consistently revealed bilateral adrenal atrophy, leading to the conclusion that the adrenal glands’ inability to respond to surgical stress was the cause of death [5–7]. The resultant concern about postoperative adrenal crisis in patients on GCs led to the routine use of high-dose perioperative GC replacement in clinical practice.

Currently, there is little high-quality evidence supporting routine perioperative use of high-dose GCs [8–11]. While underdosing perioperative GCs may place patients at risk for cardiovascular collapse, high doses carry a risk of hyperglycemia, hypertension, opportunistic infections, bone loss in a state of immobility, venous thromboembolism, and poor wound healing [12–14]. This review outlines the key physiologic aspects of the stress response to surgery, the effect of different forms of GCs on the HPA axis, the evidence for perioperative GC administration, and our personalized approach to perioperative management in adults with GC-induced AI.

## Regulation of the HPA Axis Perioperatively

**Figure 1.**
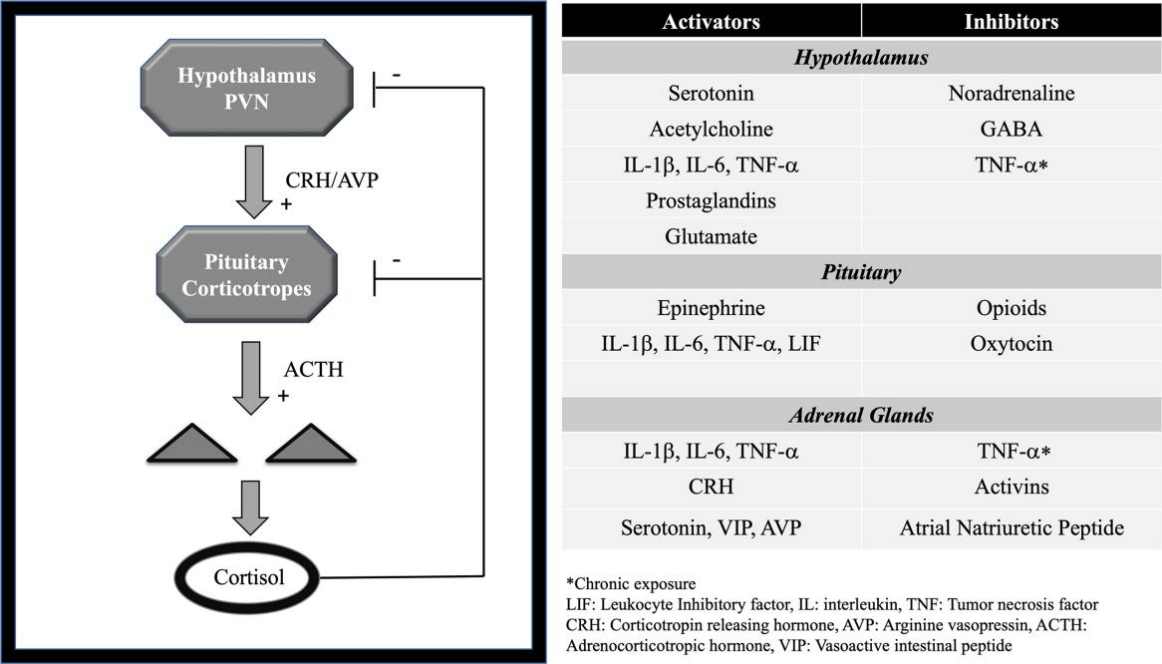
The hypothalamic-pituitary-adrenal axis regulation [24–27].

The HPA axis is regulated by the classic negative feedback of cortisol as well as other neurohumoral inputs including vasopressin, the autonomic nervous system, inflammation, and opioids (Fig. 1). This results in an estimated production of about 5.7 mg/m^2^ or 9.9 mg of cortisol per day in healthy adults which could increase to >100 mg during times of major stress [8, 15–17]. In plasma, the majority of cortisol (approximately 90%) binds tightly to corticosteroid-binding globulin (CBG) and loosely to albumin (∼5%); the remaining (∼5%) is the active, free fraction [18, 19]. Free cortisol binds to the glucocorticoid receptors in the target cell's cytoplasm. After glucocorticoids bind to its receptor, this complex translocates to the nucleus regulating the transcription, inducing or repressing a multitude of genes [20]. Glucocorticoid receptors are ubiquitously distributed in the body, including the hypothalamus and pituitary, where cortisol exerts a negative feedback [21]. Other rapid, nongenomic effects have been recognized over the past decades. Known mechanisms thought to mediate these actions include GC interaction with nonspecific cellular membrane receptors, cytosolic GC receptor mediating nongenomic effects, and membrane-bound GC receptors [22]. These nongenomic effects can involve activation of distinct signaling pathways that includes cAMP, with activation of multiple kinases, increase in intracellular calcium concentrations; as well as intracellular receptors that targets mitochondrial gene expression, all of these with a recognized tissue specificity [23]. These nongenomic mechanisms of action are highly relevant in the development of new drugs with higher selectivity and better side-effect profile compared to the classic GCs targeting the genomic actions.

Cortisol's actions are further regulated by enzymatic transformation of its active and inactive forms. In the kidney, colon, salivary glands, and placenta, 11-β-hydroxysteroid dehydrogenase type 2 inactivates cortisol into cortisone, protecting these tissues from overstimulation of the mineralocorticoid receptor. In most other tissues, but mainly in the liver, 11-β-hydroxysteroid dehydrogenase type 1 converts cortisone into cortisol, augmenting the GC effects when needed [28].

Multiple factors determine the degree of HPA axis activation during a surgical procedure. Individual factors, including genetics, age, sex, comorbidities, and medications, as well as perioperative factors, such as type and duration of anesthesia and operative procedure and perioperative complications, contribute to the heterogeneity seen in studies evaluating the HPA axis response [29].

Different types of surgical procedures generate different degrees of HPA axis activation [29–31]. Criteria to stratify the degree of surgical risk (Grade I-III), independent from the anesthetic risk, are summarized in Table 1 [32]. Multiple studies including a systematic review and meta-analysis of 71 studies including 2953 patients show that cortisol levels increase in proportion to the grade of surgery [29–31, 33, 34]. In Grade I procedures, no intraoperative cortisol peak is observed, whereas in Grade II procedures, peak cortisol occurs at the time of extubation and returns to baseline at 24 hours [29, 30]. With Grade III procedures, peak cortisol occurs 6 to 18 hours postoperatively, persists for 24 hours, and returns to baseline by postoperative day 5 to 7 [29–31, 33, 34]. Therefore, the maximum activation of the HPA axis occurs within the first 24 hour postoperatively, returning to baseline in <7 days even in patients undergoing Grade III operations [35, 36].

**Table 1.** **Surgical stress associated with common surgical procedures, based on the modified Johns Hopkins surgical criteria [** 32 **]**

Grade	General Characteristics	Characteristic Operations
Grade I (Minor)	Minimal to mild risk independent of anesthesia Minimal to moderately invasive procedure Potential blood loss of < 500 mL	Minor general surgical procedures (skin/subcutaneous tissue procedures, inguinal hernia repair, breast biopsy) Endoscopy (including cystoscopy, hysteroscopy, bronchoscopy, minor laparoscopy, arthroscopy) Minor gynecologic procedures (tubal ligation, dilation, and curettage) Minor otolaryngology procedures (myringotomy tubes, tonsillectomy/rhinoplasty)
Grade II (Moderate)	Moderate risk independent of anesthesia Moderately to significantly invasive procedures Potential blood loss of 500-1500 mL	Open or laparoscopic resection/reconstruction of the digestive tract; cholecystectomy Thyroidectomy Cystectomy, nephrectomy Hysterectomy or myomectomy Laminectomy Joint replacement
Grade III (Major)	Major to critical risk independent of anesthesia Highly invasive procedure Potential blood loss >1500 mL Usual postoperative intensive care unit stay with invasive monitoring	Any major orthopedic-spinal, oropharyngeal, or genitourinary repair or reconstruction Any intracranial, major vascular, or cardiothoracic procedure

To understand the perioperative HPA axis response, Udelsman et al measured blood samples every 10 minutes during and after Grade II neck operations performed with identical sedation and anesthetic regimens. Interestingly, CRH, ACTH, and cortisol levels were unchanged intraoperatively. However, there was a marked elevation in ACTH and cortisol levels during anesthetic reversal, endotracheal extubation, and immediately postoperatively [37]. In contrast, in Grade III operations, cortisol remained elevated on postoperative day 2, but there was a marked reduction in CRH and ACTH levels [38]. A recently reported significant decrease in cortisol clearance during critical illness could explain this postoperative day 2 dissociation between CRH/ACTH and cortisol levels through negative feedback [39]. Furthermore, neural regulation of the HPA axis seem to have an important role in the modulating the stress response to surgical procedures. Studies showing that epidural or local anesthesia can interrupt HPA axis activation following a surgical incision highlight its role [40, 41]. Also, the sympathetic nerves of the adrenal cortex can facilitate the ACTH response [42].

In addition to an increase in cortisol secretion, immediately postoperatively there is an approximately 30% to 50% decrease in CBG, and this reduction persists at 24 hours after surgery, resulting in elevated free cortisol levels [43, 44]. This increment in free cortisol could be associated with inflammation, given that interleukin-6 decreases CBG concentration by about half and the affinity of CBG for cortisol is reduced by neutrophil activation and fever, both common perioperatively [18, 45].

## Perioperative Symptoms of Adrenal Insufficiency and Cardiovascular Collapse

GCs have an essential role in the regulation of vascular tone, cardiac, and adrenomedullary function. In blood vessels, they have a permissive role in the action of vasoactive substances, particularly catecholamines [46, 47]. Additionally, GCs promote vasoconstriction by inhibiting endothelial production of nitric oxide and prostacyclin [17, 47], upregulating angiotensin II receptor AT1 [48] as well as increasing α-1 adrenergic receptors and norepinephrine-binding affinity in the vascular smooth muscle cells [47, 49]. Accordingly, during an adrenal crisis, there is a markedly reduced vascular tone resulting in hypotension that can eventually become refractory to vasopressors.

In the heart, GCs increase contractility by increasing the expression of genes that regulate intracellular calcium concentrations [50]. Studies in AI reported reduced stroke volume and cardiac index with increased systemic arterial resistance independent of changes in serum sodium and potassium levels [51, 52]. Additionally, AI has been associated with high-output heart failure that can resemble the septic shock-associated loss of vascular tone [52, 53]. These 2 hemodynamic profiles associated with hypotension depends on the degree of fluid resuscitation and the time the right heart catheterization is performed [52]. Patients with AI may have prolongation of the PR interval, which can reach a first-degree AV block or worsen the underlying AV conduction abnormalities [54]. These effects on cardiac output and the conduction system in AI can be reversed by GC replacement [55–57].

Adrenal medullary development and function are highly dependent on the presence of adequate amounts of cortisol [58, 59]. The expression and activity of phenylethanolymine N-methyltransferase, the enzyme that converts norepinephrine into epinephrine, requires a high intra-adrenal cortisol concentration that is maintained by the adrenoportal system between the cortex and the medulla [60]. Glucocorticoids also inhibit catechol-O-methyl-transferase, resulting in decreased clearance of epinephrine [47]. Therefore, the adrenomedullary function can become severely compromised in AI with markedly low levels of epinephrine at baseline and incomplete response during stress [61].

Beyond cardiovascular instability and collapse, the symptoms of AI in the perioperative period can be subtle and may be missed due to their similarity to common postoperative complaints including anorexia, fatigue, nausea, vomiting, abdominal pain, muscle cramps, weakness, dizziness, and lethargy [62]. The clinical team must be aware of GC withdrawal symptoms that may occur in patients on chronic GC on a fast perioperative taper; these patients who may experience AI-type symptoms despite being maintained on supraphysiological GC doses [63, 64].

## The HPA Axis in Patients Using Glucocorticoids

### Pharmacological Properties of Glucocorticoids

Glucocorticoids have historically been classified as short, intermediate, and long-acting according to their biological half-life (Table 2). However, GCs have many other variable pharmacologic properties, including administration route, potency, and affinity for the GC receptor, resulting in a heterogeneous group of drugs with different potentials to suppress the HPA axis.

**Table 2.** **Pharmacological properties of frequently used glucocorticoids [** 65–68 **]**

Classification	Equivalent Glucocorticoid Dose (mg)	Glucocorticoid Activity	Mineralocorticoid Activity	Biological Half-Life (hours)	Plasma Half- Life (hours)	Bound in Plasma (%)
Short Acting						
Hydrocortisone	20	1	1	8-12	1.3-2.3	90
Cortisone acetate	25	0.8	0.8	8-12	0.5	—
Deflazacort	5	4	1	<12	1.3-1.9	40
Intermediate-Acting						
Prednisone	5	4	0.8	12-36	2-4	75
Prednisolone	5	4	0.8	12-36	2-4	95
Methylprednisolone	4	5	0.5	12-36	2-4	78
Triamcinolone	4	5	Negligible	12-36	0.5	68
Long-Acting						
Betamethasone	0.6	25-30	Negligible	36-72	5	64
Dexamethasone	0.375-0.75	30-40	Negligible	36-72	3.5-5	68

In general, the absorption rate after oral administration is rapid (30-45 minutes) and similar among different preparations with bioavailability ranging from 60% to almost 100% [69,70]. Intramuscular absorption is rapid, whereas the absorption after subcutaneous injections is slightly slower depending on the amount of adipose tissue present [71]. Systemic absorption from other formulations such as inhaled, topical, ophthalmic, buccal, or rectal administration is lower, but all can potentially suppress the HPA axis [72]. Topical and mucosal absorption of GCs depends on the integrity of the skin/epithelial barrier, which is modified by inflammation and influenced by the thickness of skin [72]. Certain GCs such as Triamcinolone after intra-articular and epidural injection are slowly absorbed resulting in sustained supraphysiological concentration. Oral budesonide has a high first-pass effect in the liver where about 90% is inactivated. Despite this, HPA axis suppression and cases of adrenal crises have been reported [73, 74].

### Incidence of Postoperative Adrenal Insufficiency

Postoperative AI in patients on chronic GCs is rare [8, 17, 75]. However, establishing the true incidence is challenging. In many cases, hypotension that resolves in response to GCs, in the absence of an alternative explanation, has been used as the criteria to establish the diagnosis of AI. A 1994 review of 57 cases of patients on chronic GCs who died possibly from postoperative adrenal crisis found confirmation in only 3 cases [8]; this and other reports [33, 76] indicate that post-op AI may not be as common as initially thought. In some instances, alternative etiologies such as major blood loss, anaphylaxis, or sepsis could have explained the postoperative hypotension. However, the incidence of postoperative AI may be underreported since reporting such statistics may be undesirable for hospitals. There is a clear need for further studies in this subject.

### Risk for HPA Axis Suppression in Different Clinical Scenarios

The risk for HPA axis suppression in relation to GC exposure depends on multiple factors including the type of GC, ability of the drug to reach the systemic circulation, dose, and duration of treatment. However, the exact dose and duration that results in clinically significant AI has been a matter of debate [64, 77–80]. In general, systemic treatments, long-acting GCs, higher doses, longer duration of therapy, higher potency, nighttime administration, multiple cycles of treatment, and multiple daily doses carry a higher risk for HPA axis suppression and AI [64, 77–82]. Cushingoid features should alert clinicians of the presence of HPA suppression. Stopping chronic GCs close to an operation may have a significant impact on the risk for postoperative AI. Regardless, there is a poor correlation between biochemical HPA axis suppression and clinical outcome (AI) [77–80, 83, 84].

#### Oral glucocorticoids

The daily requirement of a patient with AI to maintain basal, nonstressed body functions is approximately 4 to 5 mg prednisone equivalent per day. To stratify the risk of HPA axis suppression, GC intake may be divided into low dose (<5 mg prednisone equivalent), medium dose (5-20 mg), and high dose (>20 mg) per day. The duration of GC exposure can be considered as short-term (<1 month), intermediate-duration (between 1-3 months), and long-term (>3 months). However, these subclassifications have a role in perioperative management of patients if there is a plan to stop GCs before the procedure. Otherwise, most patients should receive a short course of GCs perioperatively based on the level of surgical stress and then return to their basal dose as they recover.

Low-dose GCs administered in the morning, for any duration of time, seem to have a low risk of causing clinically significant AI. In a study of 50 patients on long-term prednisone (mean duration approximately 4 years), those receiving <5 mg/day showed a normal or near-normal response to the Cosyntropin stimulation test (CST) without AI-related events [85]. Accordingly, we do not recommend additional workup in patients taking <5 mg prednisone equivalent per day in the morning beyond the continuation of their glucocorticoids and monitoring them for any signs and symptoms of AI. However, it is also important to consider the cumulative effect of previous glucocorticoids in patients in whom the dose of GCs has been tapered to less than 5 mg prednisone equivalent per day at the time of their surgical evaluation. Such patients should receive a short course of parenteral GC therapy when unable to take their daily GC dose.

High-dose GCs taken for short-term frequently cause HPA axis suppression although rarely clinically significant AI. Carella et al reported that a short course of high dose of prednisone (40 mg 3 times per day for 3 days followed by a taper during the subsequent 4 days) resulted in transient HPA axis suppression [86]. HPA axis recovery based on a CRH and CST was seen 1 week after treatment. Similarly, Spiegel et al showed that prednisone at doses of 40 mg/m^2^ to 100 mg/m^2^ daily for <1 month could result in HPA axis suppression with recovery in <1 week and no clinically significant AI events. In the authors’ experience, it is common to see random and morning cortisol levels <5 µg/dL within a few days after a 6-day course of methylprednisolone (“Medrol pack”) or short courses of “pulse doses” of methylprednisolone (250-1000 mg). However, neither the literature review nor our observations have found clinically significant AI-related events in such patients. Therefore, the duration of therapy seems to have a greater impact than the dose on HPA axis suppression. Indeed, all historically reported adrenal crises have been in patients on chronic GC therapy. Therefore, we do not recommend any action outside of routine perioperative monitoring in patients who have been on GCs for <4 weeks prior to surgery.

Patients taking 5 mg or more of prednisone equivalent per day for >1 month may have variable degrees of HPA axis suppression [87]. The risk of HPA axis suppression is higher in patients taking higher doses for a longer duration. Such patients should not stop GCs prior to surgery without further evaluation of the HPA axis. The majority of these patients can stay on their current dose of GC perioperatively and can be administered a short course of intravenous GC postoperatively until they resume their oral intake (see therapy section).

##### Other variables that influence the risk of HPA axis suppression

The time of the day of GC administration can influence the risk of HPA axis suppression. In 8 healthy subjects who received 0.5 mg of dexamethasone at midnight for 2 consecutive nights, there was a more prolonged HPA axis suppression compared to those who received the same regimen at 8 Am or 4 Pm [88]. The administration of multiple GC doses throughout the day carries a higher risk for HPA axis suppression than the same total daily dose taken once a day [87, 89]. Myles et al in a crossover design compared cortisol levels after giving patients a single dose of prednisolone at 10 Am for 8 weeks vs the same total daily dose divided as half at 10 Am and the other half at 10 Pm for 8 weeks, returning to a single daily dose after [89]. Cortisol levels were significantly lower in patients during the time the dose was split compared to when patients received a single daily dose of prednisolone. Alternate-day (morning) GC regimens are associated with a lower incidence of iatrogenic Cushing syndrome and HPA axis suppression [64, 90].

There is a higher risk of AI-related clinical events if GCs are stopped closer to the date of surgical procedure, as the HPA axis needs time to recover [34]. In patients with inflammatory bowel disease (IBD) taking up to 180 mg/day of prednisone for 2 to 23 months, the ones who stopped prednisone >2 months prior to surgery had the lowest risk of developing AI-related hypotension [76]. This retrospective study should be interpreted with caution since, when adjusted by disease severity, no differences in hypotension were observed.

#### Nonoral glucocorticoid use

##### Intra-articular and epidural injections

The risk of AI with intra-articular GCs remains largely unrecognized and likely underestimated [72]. Both the absorption and the ability to suppress the HPA axis are proportional to the GC dose, half-life, solubility, vascularization of the synovium (increased during inflammation), number of joints injected, and frequency of injections [91–93]. In a meta-analysis, the intra-articular and the oral route carried the highest risk for HPA axis suppression [81]. In a small randomized controlled study that used a single fixed dose of 80 mg of methylprednisolone knee injection vs placebo, 25% of the patients receiving GC developed HPA axis suppression between week 2 and 4 after injection, and then returned to baseline [94]. When multiple joints are simultaneously injected, HPA axis suppression takes longer to recover. Habib et al reported that injecting 80 mg of methylprednisolone acetate in both knees simultaneously resulted in HPA axis suppression in 60% of the patients by week 1, 30% to 35% between week 2 and 4, and 10% of patients at week 8 [95]. Epidural GC injections can also result in rapid and prolonged HPA axis suppression. A single dose of triamcinolone resulted in suppression of the HPA axis within 1 week, slowly returning to baseline in about 4 to 12 weeks [96]. We receive a substantial number of referrals for AI of unknown etiology where previous GC injections were not elicited from the history. Until better quality data is available, we recommend evaluating the HPA axis in patients who have received 3 or more GC injections within 6 months before surgery [97].

##### Inhaled and intranasal

Inhaled GCs are absorbed systemically by the pulmonary vasculature with a smaller fraction swallowed and absorbed by the gastrointestinal tract [72]. In a systematic review in patients with asthma using only inhaled GCs, higher risk of HPA axis suppression was seen with high doses of fluticasone equivalent (>1000 mcg/day), compared to medium (200-1000 mcg/day) and low doses (<200 mcg/day), resulting in AI in 18.5%, 5.4% and 1.5% of patients, respectively. When analyzed by the duration of treatment, long-term use (>1 year) had a higher prevalence of AI compared to medium (1 month to 1 year) and short term (<1 month); duration of treatment was associated with abnormal HPA axis in 20.3%, 9.0%, and 1.3% of patients, respectively [81]. Another meta-analysis showed that the risk of basal cortisol suppression was higher with fluticasone compared to other inhaled steroids at the same equivalent dose [98]. The literature on AI from intranasal GCs is scarce. Intranasal fluticasone preparations in the United States are sold in quantities of 27.5 mcg and 50 mcg per spray. However, fluticasone nasal sprays purchased outside the United States may have higher strengths; authors have treated a patient who purchased fluticasone abroad at a strength of 250 mcg per spray resulting in GC-induced AI.

##### Topical glucocorticoids

Topical GCs are classified based on potency into 7 classes and 3 subgroups: ultra-high (Class 1-3), moderate (Class 4-5), and low (Class 6-7) potency corticosteroids. Topical GCs can be absorbed systemically through the skin depending on the surface area of application, location applied, and skin thickness and integrity of the skin (ie, ulcerated, injured, or inflamed skin results in greater absorption) [72]. Suppression of the HPA axis and AI can occur with the most potent topical GCs, clobetasol propionate 0.05% (Class 1), and betamethasone dipropionate 0.05% (Class 2). Iatrogenic Cushing syndrome accompanied by HPA axis suppression has been reported in adults with doses of approximately 33 to 100 g/week of clobetasol propionate and 49 to 80 mg/week of beclomethasone dipropionate [99–101]. A fingertip of GC in a 5 mm nozzle tube is about 0.5 g (slightly lower in women) [102]. HPA axis suppression may appear within days of the use of high potency topical GC and the duration of suppression is variable from weeks to months [99–101]. A 2002 literature review identified 1 fatality due AI in a child on whom large amounts of topical betamethasone was applied for generalized psoriasis [103]. Therefore, patients using potent topical GCs for prolonged periods of time or in combination with systemic GCs need to be cautious about the development of AI.

##### Other routes

The ophthalmic and rectal routes of steroid application have been associated with HPA axis suppression in small studies or case reports. Ophthalmic solutions, when used excessively and for prolonged periods, can be systemically absorbed resulting in iatrogenic Cushing syndrome and HPA axis suppression [104]. Rectal GCs (enemas) have been associated with systemic absorption and HPA axis suppression particularly when the rectal mucosa is injured, inflamed, or ulcerated [105].

### Special Situations

Many GCs are metabolized via cytochrome P450 3A4 (CYP3A4). Therefore, inhibition of this enzyme results in risk of higher GC levels and HPA axis suppression. Multiple medications are known to inhibit CYP3A4; for example, ritonavir, a protease inhibitor, has a known interaction with inhaled fluticasone [92, 106, 107]. When taken concomitantly with ritonavir, fluticasone propionate at doses as low as 500 ug/day for 2 months can result in iatrogenic Cushing syndrome [108]. Cobicistat, an HIV drug enhancer which also inhibits CYP3A4 when used with ritonavir, can make this interaction more severe [109]. Beclomethasone dipropionate is metabolized to a lesser degree by CYP3A4 and therefore could be an alternative to fluticasone when a combination with CYP3A4 inhibitors is required. Conversely, when CYP3A4 inducers are used, GCs metabolism increases, which may result in increased risk of perioperative AI in patients on a fixed GC dose. Some examples of CYP3A4 inducers include antiepileptic drugs, rifampin, and mitotane [92]. A list of drugs affecting the HPA axis is included in Table 3 [92, 110, 111].

**Table 3.** **Common drugs affecting the hypothalamic-pituitary adrenal axis [** 92, 97, 110, 111**]**

Drugs Affecting CRH and ACTH Synthesis or Secretion	
Opioids (morphine, oxycodone, fentanyl, tramadol, methadone, heroin) Benzodiazepines (midazolam, oxazepam, alprazolam, diazepam) Megestrol Acetate Medroxyprogesterone	
**Drugs Affecting Cortisol Synthesis**	
Azoles (Ketoconazole/Levoketoconazole, Posaconazole, fluconazole) Etomidate Metyrapone, Aminoglutethimide Trilostane	
**Drugs Affecting Cortisol Action**	
Mifepristone	
**Drugs Affecting Cortisol Metabolism**	
Cytochrome P450 3A4 Inhibitors (Decrease Cortisol Metabolism)	Cytochrome P450 3A4 Inducers (Increase Cortisol Metabolism)
Protease inhibitors (darunavir, indinavir, lopinavir, nelfinavir, telaprevir, ritonavir)	Antiepileptic drugs (phenytoin, fosphenytoin, phenobarbital, primidone)
Cobicistat, Azoles (itraconazole, voriconazole), clarithromycin, Grapefruit juice, mifepristone	Carbamazepine, rifampin, and mitotane
Calcium channel blockers (verapamil, diltiazem), amiodarone, cimetidine, conivaptan, erythromycin, and imatinib*^b^*	Nafcillin, rifabutin, bosentan, dorafenib, efavirenz, rifabutin, St John's wort*^a^*

a Less potent inducers.

b Less potent inhibitors.

Opioids such as morphine, oxycodone, fentanyl, tramadol, methadone, and heroin have been increasingly recognized as cause of GC-induced AI [112]. Li et al reported a prevalence of about 9% of AI in chronic opioid users (mean duration of 60 months) leading them to suggest that adrenal function should be monitored in patients taking > 20 mg of morphine equivalents per day [113]. Benzodiazepines such as midazolam, oxazepam, alprazolam, and diazepam can also affect the HPA axis at the hypothalamic level [114–116]. Other drugs such as megestrol acetate and medroxyprogesterone acetate have GC activity with potential for HPA axis suppression [92]. In summary, careful review of the preoperative medications aimed at identifying drugs causing HPA axis impairment is critical. Recently, in the United Kingdom, a National Patient Safety Alert was issued stressing the importance of patients on chronic GCs to carry an emergency card to raise the awareness of the surgical team for appropriate perioperative GC management [117].

## Evaluation of Adrenal Function in Patients Using Glucocorticoids

Patients should undergo HPA testing if there is a plan to stop GC before surgery (see therapy section). Morning and random (during stress) serum cortisol levels and the CST are the most commonly used tests for the evaluation of the HPA axis. Despite some studies pointing toward suboptimal sensitivity of the CST (high false negative), and even though the idea of assessing the entire HPA axis (using insulin tolerance or Metyrapone test) vs the adrenal response to exogenous ACTH analog is attractive, the authors are not aware of any reports of patients who passed the CST and suffered cardiovascular collapse perioperatively [118–122]. The authors have taken care of a large number of patients with pituitary disorders who underwent surgery without GC coverage based on passing the CST. However, the CST may be unreliable in the first few weeks following an acute hypothalamic or pituitary event where the adrenal gland has not undergone enough atrophy [122–124].

The laboratory evaluation of AI usually starts with obtaining a morning (7 Am-9 Am) serum cortisol measurement. Cortisol <3 µg/dL is diagnostic of AI; measuring ACTH levels will distinguish between primary and GC-induced AI [125]. If morning cortisol levels are in the indeterminate range (3-10 µg/dL), a CST may be performed [126]. A stimulated cortisol value of >14 to 15 µg/dL at 30 minutes after 250 ug of IV or intramuscular Cosyntropin excludes AI [127–129]. These cutoff values are suggested based on newer cortisol assays that use monoclonal antibodies and liquid chromatography with tandem mass spectrometry that result in about 20% to 0% lower cortisol levels compared to >18 µg/dL using polyclonal immunoassays [127–130]. Dehydroepiandrosterone sulfate (DHEA-S) may be used to further assess the HPA axis in patients with indeterminate serum cortisol levels [131]. A minimum DHEA-S level of 54 µg/dL rather than the lower limit of the normal range for the age has been suggested. One caveat is that any GC exposure in the past may result in long-term low DHEAS levels despite normal cortisol reserve.

## Perioperative Glucocorticoid Management

### Studies Evaluating the Effects of Supplemental Perioperative Glucocorticoids

Studies over the past several decades addressing the use of supplemental perioperative GCs in those on chronic GC therapy have major limitations. This has resulted in significant challenges to standardize clinical practice. As observed by Lamore et al, low-risk patients within the same institution often received hydrocortisone ranging between 50 and >200 mg/day for similar surgical risk, demonstrating a lack of agreement among clinicians [132].

The question of how much cortisol is needed to prevent adrenal crisis is still unanswered. Based on the 3 randomized controlled trials (RCTs) [133–135], at least 6 cohort [118, 136–140], and 1 retrospective studies [141], in most cases continuing the daily preoperative GC regimen seems to be sufficient. The 3 RCTs had small sample sizes (between 17-92) and limitations, reflecting the need for additional studies in the field. Udelsman et al showed that in adrenalectomized primates who underwent open cholecystectomy, physiological GC replacement doses resulted in similar outcomes when compared to 10-fold supraphysiological doses [142]. In contrast, when a subphysiological dose (1/10 of the normal cortisol production rate) was given perioperatively, more hemodynamic instability with a higher mortality rate was observed [142]. In Table 4 we summarize the relevant clinical studies that support the notion that, in most cases, the current practice of perioperative GC administration is excessive and potentially harmful.

**Table 4.** Studies addressing supplemental perioperative glucocorticoid treatment

Source	Type of Study	Population	Perioperative Glucocorticoid Regimen	Total Dose per Day	Duration of the Protocol	Type of Surgery	Outcome
Solem 1962 [136]	Prospective	Heterogeneous (n = 78)	Group 1: A 7-day cortisone regimen for those under chronic glucocorticoid at the time of surgery vs Group 2: As needed therapy for those who stopped glucocorticoids > 4 weeks pre-op	100 mg 1 day pre-op 300-400 mg day 1 200 mg day 2 150 mg days 3-4 100 mg days 5-6 Baseline dose day 7	7 days	Various Mostly major stress	No AI in either group
Lloyd 1981 [137]	Prospective	RA (n = 61)	Group 1: Hydrocortisone once, then only if post-op hypotension vs Group 2: PRN hydrocortisone Patients in both groups were on their chronic glucocorticoid regimen	100 mg day 1	1 day	Various orthopedic procedures	PRN regimen was favored
Symbreg 1981 [118]	Prospective	Heterogeneous (n = 22)	Group 1: Controls not previously exposed to glucocorticoids Group 2: Hydrocortisone treatment if abnormal CST vs Group 3: Monitoring if normal CST*^b^*	125 mg day 1	1 day	Various Mostly major stress	No AI in either group*^a^*
Shapiro 1990 [139]	Prospective	Renal transplant (n = 13)	Group 1: Continued their daily dose of prednisone (no supplemental glucocorticoids were given)	5-20 mg	n/a	Allograft Nephrectomy	No AI with continuing daily dose of prednisone
Bromberg 1991 [138]	Prospective (Cohort)	Renal Transplant (n = 40)	Group 1: Same population Controls Group 2: Continue daily dose of prednisone (no supplemental glucocorticoids were given)	5-10 mg	n/a	Various surgical and non-surgical stress	No AI with continuing daily dose of prednisone
Friedman 1995 [140]	Prospective	RA, renal transplant (n = 28)	Group 1: Continued their daily dose of prednisone (no supplemental glucocorticoids were given)	1-20 mg	n/a	Major orthopedic surgeries	No AI with continuing daily dose of prednisone
Bromberg 1995 [143]	Prospective	Renal transplant (n = 52)	Group 1: Continued their daily dose of prednisone (no supplemental glucocorticoids were given)	5-15 mg	n/a	Various	No AI with continuing daily dose of prednisone
Glowniak 1997 [133]	Randomized controlled trial	Heterogeneous (n = 17)	Group 1: Daily dose + stress dose hydrocortisone vs Group 2: Daily dose + saline (placebo)	200 mg day 1 100 mg day 2 50 mg day 3	3 days	Various	No additional benefit from stress dose hydrocortisone
Thomason 1999 [135]	Randomized controlled trial Crossover	Organ transplant (n = 20)	Group 1: Daily dose + single stress dose hydrocortisone vs Group 2: Daily dose + saline (placebo)	100 mg	1 day	Gingival surgeries Minor stress	No additional benefit from stress dose hydrocortisone No AI in either group
Mathis 2004 [141]	Retrospective	Renal Transplant (n = 58)	Group 1: Stress dose glucocorticoids vs Group 2: No stress dose*^b^*	n/a	n/a	Lymphocele drainage	No additional benefit from stress dose glucocorticoids and more hyperglycemia was reported
Zaghiyan 2011 [144]	Retrospective	IBD (n = 49)	Group 1: Stress dose glucocorticoids vs Group 2: No stress dose	100 mg (incision) 300 mg day 1 Taper to prednisone 20 mg per day	4 days	Major abdominal surgeries	No additional benefit from stress dose glucocorticoids No AI in either group
Zaghiyan 2012 [145]	Prospective	IBD (n = 32)	Group 1: Patient not currently on glucocorticoid (but previously exposed during the past 12 months) received no treatment perioperatively vs Group 2: Patients currently on glucocorticoids receiving low-dose supplemental glucocorticoids perioperatively	One-third of pre-op dose (incision) One-third of the dose Q8hrs day of surgery One-fourth Q8hr day 1 One-sixth Q8hr day 2 One-fourth Q12hr day 3 Prednisone equivalent day 4	4 days	Major abdominal surgeries	No AI in either group Low-dose supplemental glucocorticoid regimens seem safe
Zaghiyan 2012 [146]	Retrospective	IBD (n = 97)	Group 1: High (stress) dose glucocorticoids vs Group 2: Low-dose supplemental glucocorticoid (see [145])	100 mg (Incision) 300 mg day 1 Taper to prednisone	4 days	Major abdominal surgeries	No AI in either group No additional benefit from high-dose stress glucocorticoids
Ayatac 2013 [147]	Retrospective	IBD (n = 235)	Group 1: Daily dose + Stress dose glucocorticoids vs Group 2: Daily dose (unless off glucocorticoids preoperatively) + no stress dose	100 mg (operating room) 300 mg day 1 Then taper	n/a	Major abdominal surgeries	One case of AI in group 1. No additional benefit or harm was seen from stress dose glucocorticoids
Zaghiyan 2014 [134]	Single blinded randomized controlled trial	IBD (n = 92)	Group 1: Stress dose hydrocortisone vs Group 2: Hydrocortisone equivalent to oral daily dose	100 mg (incision) 300 mg day 1 225 mg day 2 150 mg day 3 100 mg day 4 20 mg Prednisone	5 days	Major abdominal surgeries	No additional benefit from stress dose glucocorticoids

Excluded studies where both chronic glucocorticoids and supplemental treatment were not provided [33, 148].

AI, adrenal insufficiency; CST, cosyntropin stimulation test; IBD, inflammatory bowel disease; n/a, not available; RA, rheumatoid arthritis.

a 125 mg resulted in higher cortisol levels than nonexposed.

*^b^* Unclear if patients were receiving or stopped chronic glucocorticoids at the time of surgery.

The first RCT studied 17 patients on prednisone 5 to 60 mg/day for a duration of 2 months to 20 years [133]. All patients had abnormal CST. These patients were kept on their daily dose of prednisone and randomized to saline injections vs stress doses of 200 mg of hydrocortisone with a gradual taper over 3 days. No adrenal crisis was observed in the group on the daily dose of GC and placebo [133]. In the second RCT, 20 solid organ transplant patients receiving maintenance doses of prednisone (5-10 mg/day) were randomized to continue their chronic GC regimen vs additional 100 mg of hydrocortisone during gingival surgery under local anesthesia. No significant difference in hypotension or signs of AI was observed in either group [135]. Lastly, a single blinded RCT included 92 patients with IBD undergoing major colorectal surgery who were randomized to receive either hydrocortisone 100 mg every 8 hours. followed by a taper, or IV hydrocortisone equivalent dose of the presurgical GC regimen [134]. As with the previous RCTs, there was no evidence of hemodynamic benefit from supplemental stress dose GCs.

The retrospective and nonrandomized prospective studies support the RCTs’ results. In kidney transplant patients on an average prednisone dose of 15.5 mg/day for >84 days, adding stress dose GCs to their chronic GC regimen did not provide additional hemodynamic benefit during minor surgical procedures but increased the risk of hyperglycemia [141]. In another study, 40 renal transplant patients on GCs for at least 3 months were admitted to the hospital for various reasons (sepsis, metabolic abnormalities, surgery). They continued their daily prednisone regimen of 5 to 10 mg/day without supplemental treatment. When comparing this group to the same population of patients with renal allograft admitted to the hospital for various reasons during the same period of time, there was no increased mortality, length of hospital stay, or adrenal crises [138]. Other groups consistently demonstrated a lack of benefit of stress dose GCs in patients with IBD undergoing major abdominal surgery [76, 144–147].

In general, the literature supports the concept that continuing the daily GC dose in patients suspected to have GC-induced AI is sufficient to prevent adrenal crises perioperatively. In fact, in the only 3 confirmed postoperative adrenal crisis-related deaths, chronic supraphysiological GC doses were stopped before surgery [8]. Based on the level of surgical stress, parenteral GC treatment in patients who cannot tolerate oral intake is reasonable with a gradual return to daily GC dose once daily intake is resumed (Table 5).

**Table 5.** Perioperative treatment regimens suggested for patient with glucocorticoid-induced AI

Regimen	Degree of Surgical Stress	Glucocorticoid Regimen
Patients currently on glucocorticoids	Garde I Minor	Continue daily dose of glucocorticoid 25 mg of IV hydrocortisone at induction if not able to tolerate PO Resume oral daily preoperative glucocorticoid regimen
	Grade II Moderate	Continue daily dose of glucocorticoid 25-50 mg of hydrocortisone IV at induction 15-25 mg hydrocortisone every 6 hours. until PO is tolerated and hemodynamically stable*^a^* Resume oral daily preoperative glucocorticoid regimen
	Grade III Major	Continue daily dose of glucocorticoid 50 mg of hydrocortisone IV at induction 25 mg of hydrocortisone IV every 6 hours on day 1 and until hemodynamically stable, then 15 mg IV every 6 hours until PO is tolerated*^a^* Resume oral daily preoperative glucocorticoid regimen
Patients who stopped or plan to stop glucocorticoids before surgery		Assess HPA axis in patients with intermediate to high risk (see Table 4) The closer the date of discontinuing glucocorticoids before surgery, the higher the risk of AI Treat based on the degree of surgical stress in those who have abnormal HPA axis
Adrenal crisis		100 mg of hydrocortisone IV (IM if no IV access) 50 mg every 6 hours until hemodynamically stable and then taper*^a^* Taper depending on clinical response-intravenous fluids (normal saline), dextrose 5% if hypoglycemia

These recommendations include the authors’ personalized approach to perioperative management in patients with glucocorticoid-induced AI.

AI, adrenal insufficiency; HPA, hypothalamic-pituitary-adrenal; IM, intramuscular; IV, intravenous; PO, by mouth.

a Some experts favor continuous glucocorticoid infusion.

### Glucocorticoid “Coverage”

There have been a large number of supplemental perioperative GC regimens recommended over the past decades [6, 8, 11, 17, 66–156]. Some of these recommendations were based on estimated cortisol production rates during stress and others empirically based. Simultaneously, experts in the field have consistently voiced their concerns about the excess amount of GC coverage, which may be harmful (Table 6). Awareness of the lack of evidence to support the use of high-dose perioperative GCs and the associated risks has resulted in a trend to recommend lower doses and shorter duration of these protocols [12–14]—from >300 mg of hydrocortisone [118] to about 100 to 200 mg/day [11, 66, 70, 150, 156] and shorter tapers over <7 days—without increased mortality.

**Table 6.** Review articles that commented on perioperative glucocorticoid treatment regimen

Source	Year	Comments from the Authors	Conclusions on Treatment
Kehlet [151]	1975	“Glucocorticoid should only be given in a necessary and adequate dose” to avoid side effects	Most regimens are founded on empirical basis
Salem [8]	1994	The risk should be individualized based on the glucocorticoid preoperative dose, duration, and type of surgery	We are giving too much glucocorticoids
De Lange [157]	2008	There is no evidence to support excessive dosing (>200 mg hydrocortisone equivalent/day) or extensive duration in uncomplicated cases	We are giving too much glucocorticoids
Marik [158]	2008	“Stress doses are not routinely required as long as the patient continues their usual daily dose of glucocorticoids”	We are giving too much glucocorticoids
Fleager [155]	2010	“There are no evidence-based treatment guidelines that provide firm recommendations for the administration of perioperative steroids”	No evidence for current practice
Kelly [159]	2013	Based on the existing evidence, patients on long-term glucocorticoids do not require the once-standard high doses; just continue their maintenance doses perioperatively. Treat refractory hypotension with rescue doses of steroids	We are giving too much glucocorticoids
Hicks [160]	2015	“Current prescribing practices are highly variable, likely because of a lack of randomized controlled data and a wide range of preoperative treatment regimens”	“Recent data suggest that additional corticosteroid supplementation in the perioperative period may be unnecessary and may serve only to increase the risk of poor wound healing and infectious”
MacKenzie [161]	2016	“Despite little evidence for this practice (supraphysiological supplemental perioperative glucocorticoids), few have challenged this treatment paradigm”	“With few exceptions, the use of supraphysiologic glucocorticoid therapy for adults with presumed adrenal insufficiency due to exogenous glucocorticoid use should be regarded as unnecessary”
Liu [156]	2017	“Recommendations in major textbooks are confusing, inconsistent, and lacking in class A or B evidence”	There is no universal agreement regarding dose, duration, or regimen of supplemental glucocorticoid
Groleau [9]	2018	“It is not possible to conclude that perioperative administration of corticosteroids, compared to placebo, reduced the incidence of adrenal insufficiency”	Providing the daily maintenance dose without supplemental glucocorticoids may be sufficient
Khazen [162]	2018	“We found no evidence to support the use of supraphysiologic dose of glucocorticoid therapy provided the patient receive their usual dose of glucocorticoid preoperatively”	“A well-designed, large multicenter RCT is warranted”
Chilkoti [66]	2019	“There are no dogmatic guidelines regarding perioperative “stress dose” of steroids in patients on chronic steroid therapy; however, there is enough evidence that patients on long-term exogenous steroid therapy do not require the conventional high-dose perioperative corticosteroid, instead must be kept on their baseline maintenance dose”	We are giving too much glucocorticoids
Manou-Stathopoulou [163]	2019	“Clinical trials exploring glucocorticoid supplementation have provided conflicting data, reflecting the lack of understanding of the cortisol biology during the perioperative period”	More personalized targeted therapies are needed
Seo [11]	2021	Many clinical trials have low level of evidence, lack of power, without clear criteria for AI that results in high variation in the recommendations	No evidence for current practice
Laugesen [10]	2021	“Current evidence indicates substantial variation regarding risk and course of glucocorticoid-induced adrenal insufficiency … more research is needed to refine the diagnosis and to support evidence-based clinical decision-making”	No evidence for current practice

Many review articles discuss the topic and make recommendations without specific comments about the current practice.

AI, adrenal insufficiency; RCT, randomized controlled trial.

In a study by Arafah [164], 20 mg of oral hydrocortisone 2 to 4 hours. prior to surgery resulted in a baseline cortisol of 14.8 ug/dL in those with central AI. The administration of 25 mg of hydrocortisone IV resulted in a nadir serum cortisol range of 16 to 34 ug/dL at 6 hours, which was higher with subsequent injections. In this setting, cortisol half-life was longer, volume of distribution increased, and clearance was lower in patients with AI compared to healthy individuals. This study suggests that patients with AI who are administered 20 mg of hydrocortisone 2 to 4 hours prior to intubation have a baseline cortisol level comparable to healthy individuals with an intact HPA axis. Subsequently, providing 25 mg of IV hydrocortisone every 6 hours for 24 hours, followed by 15 mg every 6 hours or 24 hours, resulted in no adverse events or symptoms suggestive of AI. This study did not include patients with GC-induced AI, but the same concepts may be applied as most subjects in this study had secondary AI. Additionally, cardiac surgery procedures were not included. The results are in agreement with the trend that our current practices provide higher perioperative GC doses than needed [165]. Considering this, 15 to 25 mg of hydrocortisone IV every 6 hours (60-100 mg/day) should provide enough perioperative coverage for even moderate to major operations in patients suspected to have GC-induced AI. Others have shown that continuous hydrocortisone infusion provides more stable cortisol concentrations during major stress without significant peaks and troughs that may be seen with intramuscular or IV administrations. This approach requires an additional IV infusion line, and no data indicate that continuous hydrocortisone infusion prevents adrenal crisis or is associated with lower adverse events compared to intermittent hydrocortisone injections [70]. We have summarized our perioperative approach in Table 5.

There is little data in perioperative management of pregnant patients on chronic GC. Cortisol levels increase throughout pregnancy secondary to increased CBG and to the HPA axis stimulation by placental CRH. The dose of GC replacement does not usually need to be increased during the first and second trimesters, but an increase in GC dosage of 20% to 40% from the 24th week forward is generally recommended [166]. Accordingly, a 50% higher perioperative parenteral GC coverage, especially during the third trimester, and delivery seems reasonable. Careful perioperative monitoring of the hemodynamic status of pregnant women and their fetus is critical [166–168].

## Conclusions

Perioperative management of patients on GCs has been a major topic of discussion for over 70 years. It is clear that assessment of the HPA axis in patients who have stopped GC therapy before surgery is necessary. However, despite the progress in our understanding of the stress response and hormonal behavior perioperatively, there is significant heterogeneity in clinical practice in terms of GCs dosing. In most cases, excess GCs are administered, which may result in several adverse events. The current literature supports that in patients undergoing a surgical procedure, continuing the daily dose of GCs along with a short course of perioperative IV GCs based on the level of anticipated surgical stress is adequate. In most perioperative scenarios, administration of ≤100 mg/day hydrocortisone with a rapid taper to preoperative GC dose is sufficient. Close monitoring for any evidence of hemodynamic instability is fundamental. Finally, there is a need for large prospective studies to optimize the perioperative management of patients on GCs to avoid any clinically significant AI-related event and do no harm.

## Data Availability

Data sharing is not applicable to this article as no datasets were generated or analyzed during the current study.

## Abbreviations

AI: adrenal insufficiency
CST: cosyntropin stimulation test
DHEA-S: dehydroepiandrosterone sulfate
GC: glucocorticoid
HPA: hypothalamic-pituitary-adrenal
IBD: inflammatory bowel disease
RCT: randomized controlled trial
